# Healthcare middle managers` experiences developing leadership capacity and capability in a public funded learning network

**DOI:** 10.1186/s12913-018-3259-7

**Published:** 2018-06-08

**Authors:** Trude Anita Hartviksen, Berit Mosseng Sjolie, Jessica Aspfors, Lisbeth Uhrenfeldt

**Affiliations:** 1 Faculty of Nursing and Health Sciences, Nord University, Bodø, Norway; 2 Faculty of Education and Arts, Nord University, Bodø, Norway

**Keywords:** Healthcare middle manager, Leadership, Capacity, Capability, Learning network, Complexity

## Abstract

**Background:**

Healthcare middle managers (HMMs) have, as the leaders closest to clinical practice, a crucial position in healthcare today. There is broad knowledge about the demands on HMMs’ capacity, their situation in general, and the challenges this presents for the improvement of healthcare quality. There is less knowledge about how to facilitate HMMs` capacity and capability with regard to their leadership and how to handle this in a complex context. The purpose of this study was to identify and discuss the facilitation of HMMs’ development of capacity and capability for leadership.

**Method:**

A critical hermeneutic design was chosen. Data were collected through three focus group interviews with Norwegian HMMs who participated in a learning network. A user representative (from among the recipients of public healthcare), involved in the same learning network, participated in all three interviews. A qualitative interpretive approach guided the analysis.

**Results:**

The results show two main themes: 1. Trusted interaction despite organizational and structural frames and 2. Knowledgeable understanding of a complex context.

**Conclusion:**

This learning network facilitated HMMs` development of capacity and capability for leadership. The development included a combination of understanding the complex context, knowledge, trust, and confidence. The approaches in the learning network were based on transformative learning, coherence, reflection, discussion, repetition, knowledge sharing, and short lectures. These approaches can be recommended for the facilitation and support of HMMs.

**Electronic supplementary material:**

The online version of this article (10.1186/s12913-018-3259-7) contains supplementary material, which is available to authorized users.

## Background

Healthcare middle managers (HMMs) are, as leaders, closest to everyday clinical practice and have a crucial role in translating top-level policies, strategies, and means, to achieve practical improvements in healthcare delivery [1–3]. Turnover and a shortage of personnel, engagement, motivation, and accomplishments in the workplace are all factors closely associated with leadership and management [2–4].

This study involves HMMs` development of capacity and capability for leadership, to manage the complex context they are a part of, and how this developmental process can be facilitated. Capacity is understood as the individual features possessed by HMMs, such as technical expertise, creative thinking skills, social skills, and organizational understanding [5]. Illeris [6] defines learning as the process that changes a person’s capacity. Capability is, on the other hand, understood as what HMMs are able to do, such as to identify and define problems and to establish and manage an evolving context [5].

This study’s research question is as follows: How did HMMs, who participated in a learning network, experience that this participation contributed to the development of capacity and capability for leadership in a public funded healthcare system characterized by high complexity?

### Healthcare middle managers

Healthcare management is traditionally characterized by strategic planning and implementing concrete tasks in a leadership structure based on hierarchical and linear leadership styles [7]. Lately, this type of leadership has been criticized as reductionist and limiting due to a lack of ability to account for highly complex, interrelated, relationship-driven organizations [1, 7–9]. An example of hierarchical and linear leadership styles is described by the full range leadership model, transactional leadership. Transactional leadership relates to external motivation, contingent reinforcement, guidelines, command and control. The full range leadership model also includes two alternative leadership styles: transformative leadership and laissez-faire. Whereas transformative leadership is based on inspiring creativity, flexibility, and appealing to inner motivation, laissez-faire describes absent, or passive, leadership [10]. While research previously looked for the best leadership style, present research recommends flexibility among leadership styles as different leadership styles evoke various responses [1, 11].

The importance of HMMs` capacity and capability for leadership has been less recognized in healthcare [2]. Traditionally, HMMs have primarily focused on more visible, clinical tasks and therefore their leadership actions were in addition to, and often overshadowed by, their clinical workload [2, 12, 13]. It was expected that leadership would be self-taught, learned while working [14]. HMMs have possessed a clinical background, with limited capacity and capability for leadership, both regarding qualifications, experience, and support [2]. Several studies clarify that it is necessary to improve leadership education in healthcare and to develop HMMs` capacity [2, 3, 12, 14, 15].

A changing complex context demands HMMs with new and increased knowledge [1, 7, 12], including technological [1, 2, 7, 13], socio-cultural [1, 13], economical [1, 2, 16], and political knowledge [1]. The increased complexity makes HMMs more dependent on skills such as communication, negotiation, implementation, analysis [1, 17], developing strategies [13], problem solving, leadership [2, 16], risk managing, and networking [12].

There is thus broad knowledge about the roles HMMs are anticipated to fulfill. There is less knowledge about how to acquire these specific competencies, within a complex and changing organization [9, 12, 15, 18]. Dickson [3] suggests that present leadership should be understood through complexity theory.

Complexity theory explains healthcare organizations as complex adaptive systems (CAS) [7, 19, 20]. This understanding implies that microsystems are the core of all healthcare services [21]. The microsystems consist of individual interconnected agents who acts in unpredictable ways [22, 23]. CAS have been criticized for objectifying human organizations. Complex responsive processes (CRP) are an alternative understanding in complexity theory, describing organizations as processes of human interactions [7, 23]. The complex context in this study is understood in relation to both the theory of CAS and CRP. The purpose is to identify and discuss the facilitation of HMMs` development of capacity and capability for leadership.

## Method

This study was guided through a critical hermeneutical perspective [24–27]. This methodological foundation includes Habermas` concept and understanding of a lifeworld. HMMs` lifeworld is, in this study, understood as a cultural horizon, where HMMs interpret and understand through concrete experiences and where values, norms, and language are important control mechanisms. It is understood that the participants` lifeworld is colonized by the system, which is a process that could be balanced by the participant’s reflection and critical questioning of the context of meaning, patterns of interpretation, creation of norms, and social interactions [27]. The study searches to accentuate when theoretical statements represents changeable dependent relationships [26].

### Design

The study occurred in a learning network in a rural part of northern Norway. The network was related to publicly funded healthcare. A learning network is understood as organized competence development across limited professional, or organizational, borders with the purpose of increasing knowledge and shared experience [28]. This learning network focused on quality improvement in healthcare. Learning networks that consider quality improvement, quality improvement collaboratives, are central to current international strategies to improve healthcare. A quality improvement collaborative focuses on areas in healthcare with large variations or gaps between best and current practice. A collaborative is supported by clinical experts and experts in quality improvement and involves multi professional teams from multiple sites. Such collaboratives are structured by a model of improvement, which emphasizes clear and measurable targets, data gathering, and small-scale testing of changes. The collaborative process involves structured activities in a given time frame. The purpose is to advance improvement, exchange ideas, and share experiences [29]. It has been confirmed that learning networks stimulate organizational learning better than traditional approaches, but there is a need for more empirical knowledge to build the theory in this area [9, 29].

There are different pedagogical approaches to learning based on each of five main learning theories: behaviorist, cognitivist, constructionist, humanist, or social learning [30]. The choice of theoretical approach to learning will influence HMMs` development of capacity and capability differently, as their applicability depend on the learning situation. The theoretical perspective of HMMs` development in this learning network is inspired by Illeris’ [6] perspective: transformative learning. Illeris [6] combines a variety of learning theories into a comprehensive framework, specifically aligned to adult learning [6]. This framework explains all learning as both individual and social. The individual receives impulses through social interaction, which are incorporated by internal interpretation and acquisition. It has been suggested that transformative learning involves changes in the learners` meaning perspectives, as a result of critical reflection, open discourse, and the implementation of a new understanding in practice [31].

This study’s learning network was established in 2012 and consisted of 54 participants, who met 3–4 times yearly in order to 1. share development of leader and improvement knowledge, 2. receive guidance in the practical performance of improvement practices, and 3. networking. The meetings consisted of short lectures and group workshops within and across organizational borders. The meetings were located in different conference venues in the participating municipalities. The researchers` access to the network was facilitated since both the first and second researcher had participated in the network from the initial phase. The network initially organized as a project, and therefore was partly financed by the County Center for Development of Home Care Services, partly financed by the participants` organizations, and partly financed by the County Council.

The network included participants from among the recipients of public healthcare (at the time, one user representative), 40 HMMs from rural municipalities, 10 HMMs from a local hospital, 3 lecturers from a local university department, and the manager of the County Center for Development of Home Care Services. The Norwegian Knowledge Center for the Health Services had a role as the supervisor. The participating HMMs had clinical backgrounds, mainly as nurses, but there was also one social worker, three physicians, and one occupational therapist.

### Participants

The participants of the study were volunteer members from this learning network. Aside from the one user representative, their professional backgrounds were all nurses, and they all worked as HMMs. The user representative was specially invited as at the time he was the only user representative in the learning network. The purpose of the involvement was to include this important perspective to the focus groups. The involvement of user representatives in research is known to optimize validity, design, applicability, and dissemination [32, 33]. The invitations were otherwise sent as an email to all the leaders who participated in the learning network. To capture various perspectives [34], the participants were divided into one group of municipal HMMs, one group of hospital HMMs, and one group of municipal long-term HMMs. In total, twenty-six invitations were sent. Sixteen HMMs participated (Table 1), which results in a 62% participation rate. The total number of participants was 17, including the user representative.

**Table 1:** Participants` characteristics

Participants	Focus group 1 HMMs from municipal homecare services	Focus group 2 HMMs from the local hospital	Focus group 3 HMMs from municipal long-term care	Total
HMM	5	6	5	16
User representative	1	[1]	[1]	1
Total	6	7	6	17

### Data gathering

The data were gathered in December 2014, through three successive qualitative semi-structured focus group interviews [34, 35]. The first author conducted two of the interviews, while the second author conducted the third interview. The environment of the interviews was a shielded room in a restaurant, which was chosen to ensure that the participants would be undisturbed. Each interview lasted approximately one and a half hours.

The interviews addressed the participants` experience in the development of capacity and capability for leadership by participating in a learning network. The theoretical framework of complex adaptive systems (CAS) and complex responsive processes (CRP) influenced the design of the interview guide [7, 19, 20]. The questions in the interview guide were framed to stimulate dialogue and reasoning from a critical and reflective perspective [36]. The interview guide is enclosed (see Additional file 1).

The initial questions of the interviews were open-ended. The participants were asked about: 1. their experiences with the development of capacity and capability for leadership, 2. the usefulness of the learning network, 3. their capacity as a HMM, 4. how the learning network contributed in this area, and 5. other processes in their life that could be compared to the processes occurring in the network.

The participants contributed as much detailed information as they wanted. All participants, including the user representative, participated at the same premises. The participants followed up on each other’s statements in a fluent conversation. The interviewer added complementary questions to bring forward contrasts in the participants` experiences or expectations. Such questions could be: 1. can you add some examples? 2. how did this happen? 3. how did you know this? 4. what was less, or not, useful? and/or: 5. how could this be changed.

The first and second author were present for all three focus groups and alternated positions as moderator and assistant moderator. The assistant moderator had the responsibility of audio recording the focus groups and to taking notes that included body language and other visual cues, including group dynamics [35]. The recordings with notes were transcribed into verbatim text, which amounted to a total amount of 87 pages. The transcripts were generated systematically and consistently, ensuring that all verbal and nonverbal statements were documented [34].

### Data coding and analysis

The critical interpretation of this study focuses on the construction of reality, asymmetrical relations of power, ideology, autonomy, and communicative distortions. The interpretation includes both understanding and explanation and alternates between proximality and distance. At the distance level, the interpretation relates to a broader social, historical, and economic context, a problematization of what seems natural and self-evident [36].

The use of reflection and critical questioning in focus groups, including the context of meaning, patterns of interpretation, creation of norms, and social interaction, could be understood as an attempt to rationalize the participants` lifeworld and thus balance the rationalization applied by the system. Every communication process is the result of a culturally practiced preunderstanding [27]. Considering the authors` and participants` lifeworld and preunderstanding and how this has affected their understanding of complexity was thus a central part of the analysis.

Both the first and second author had a preunderstanding of HMMs based on experiences from former HMMs positions in public healthcare and participation in the same learning network. This preunderstanding involved experiences of a demanding clinical every-day setting but also the experiences of how this situation could be influenced. The preunderstanding included an understanding of HMMs` capacity and capability for leadership as diverse and often randomly accomplished.

The transcribed text from the interviews was the focus of the interpretation. The transcribed text included stories, which were described in the interview text, about the participants` experiences with the development of capacity and capability for leadership by participating in a learning network. The interviews were read several times to get a sense of the whole. The purpose of the analysis was to deepen knowledge, leading to transformative action [37]. The analysis was done manually as this was considered an important part of the hermeneutical process. Through the analysis, we searched for latent content, while being guided by critical hermeneutic principles in accordance with Kvale [34] and Alvesson and Sköldberg [36]. Latent content addresses the relationship aspect and involves the interpretation of the underlying meaning of the text, which is deeper and more critical than what is initially expressed [34].

This analysis was based on seven main characteristics: 1. the transcribed text was interpreted in a back and forth movement according to the hermeneutical circle; 2. the interpretation was ended when a good gestalt was reached without logical contradictions; 3. partial explanations were tested in relation to the global meaning; 4. the autonomy of the text was respected as the text was understood from what it stated itself about the theme; 5. the researchers had knowledge about the theme; 6. although the interpretations were not without presuppositions, the researchers were aware of how these influenced the analysis [34]. The created reality will always be understood through intersubjectivity [38]; and 7. the interpretations involved renewal and creativity beyond what is immediately given, including new differentiations and mutually relations, as the meaning in this study expanded through an abductive process [34].

The transcribed text was condensed into meaning units in a shortening process in which the core meaning was preserved (see Table 2). Then, the condensed meaning units were abstracted and sorted under higher order headings into subthemes and themes, based on the study’s purpose [34]. The conclusions of the first analysis phase were validated by the participants in a new focus group, consisting of 10 voluntary participants from all three former focus groups. The participants were here encouraged to object to the conclusions if they did not recognize their statements. The participants confirmed the trustworthiness of the results; thus, no changes were made on this basis.

**Table 2:** Illustration of the analysis process, from the text units to the subthemes and themes

HMMs experiences of developing capacity and capability to leadership		
Themes	Sub-themes	Quotations
Trusted interaction despite organizational and structural frames	Inter-departmental knowledge and trust	“...because we have the same foundation, and we know in our head what we are talking about” “We are associates, in a way”
	Increased interaction	“We have perhaps started to think, not think, but work, more similarly, more, not like he works like this in his place, but I do it differently in my place” “But, what is good is when you have been in the network, and come back, and then it is fresh in the head, and it is easy to work with those who have been there with improvement”
Knowledgeable understanding of a complex context	Reflective processes	“The network, it is thinking work, you know, reflections” “These are things that are repeated several times and that it is… for someone, you do not get everything all the time, but then it gets repeated, some of the themes”
	Theoretical explanatory models and tools	“Now we know that there is a system too” “It is useful to have theoretical knowledge about the different tools we use”
	Handling the complex and demanding context	“...before, I did much of the same things, but it was much more fragmented…” “You know, as a leader, that you need to lift your eyes, look ahead, above the daily tasks…yes, that we need to think a bit differently”

## Results

The participants were aged 34–69. The majority of the participants were women (75%). There were two men in each group, including the user representative. These are representative numbers according to the gender ratios in Norwegian Healthcare, where 84,9% of the employees are women [39]. Table 1 describes the participants` characteristics. The parentheses in focus groups 2 and 3 indicate that this is the same participant as in focus group 1.

The results are presented in two overarching themes, consistent with participant quotations. The themes are 1. trusted interaction despite organizational and structural frames and 2. knowledgeable understanding of a complex context.

### Trusted interaction despite organizational and structural frames

In this study, a recurring theme was the participants` experiences of how the learning network contributed to their development of capacity and capability for leadership as it refuted their complex context. Knowledge and trust were developed among the participants. The network, in itself, was not limited by organizational or structural frames. Participation led to increased interaction between HMMs, both internally in the individual organizations and across organizational borders. The study’s results show that participation in the learning network provided HMMs with the possibility of seeing themselves as part of a broader perspective, the patient pathways. This was described as contrasting with their experience of a normally fragmented and solitary day.

### Inter-departmental knowledge and trust

This learning network could be described as a leadership community founded on the development of knowledge and trust among the participants. This development resulted in capacity and capability for leadership based on a common consciousness of purpose, understanding, trust, and respect among the participants. The participants stated that they had developed a broader understanding, both of themselves as HMMs and in relation to other leaders from the same context and across organizational borders. Participant 1, from the municipal homecare services, explained:

*“It is, like, related to…or to the network, when we have been there several times, and you feel that you, well, know these persons…. In addition, we have become, like, a close-knit gang…”.*

Participant 2, from the municipal long-term care, said:

*“Just that, it is important that we sort of are come as far, that we as a leadership group have heard and been through the same things…because we have the same foundation, and we know in our head what we are talking about”.*

Participant 3, from the hospital, said:

*“I have become very impressed by the work performed in home care services, and in, the municipality…I respect them…I must say, I admire them…”.*

This common knowledge and trust resulted in a team understanding among the participants; they understood each other as colleagues. This understanding was explained as a contrast to their previous view of each other, which was more like competitors.

The network had become so important for some of the participants that they would prioritize participating even if it was questioned by their senior management. This was an experience especially shared by the hospital participants. They explained that the learning network was their only meeting point related to leadership, as other meeting points were focused on reporting and economic management. Participant 4, from the hospital, explained:

*“I do not acquire anything if I do not participate in this…if this is the little I get during a year...yes, then I even will pay for it myself”.*

### Increased interaction

The learning network was described as increasing both internal and interdepartmental interactions when the participants returned to their leadership positions in their normal clinical day. Participant 5, from the hospital, explained:

*“But, that is what is good, when you have been in the network, and come back then it is fresh in the head, and it is easy to work with those who have been there with improvement”.*

Participant 6, from the municipal long-term care, said:

*“I no longer “drive solo racing”, to show others what I have achieved……We have perhaps started to think, not think, but work, more similarly, more, not like he works like this in his place, but I do it differently in my place”.*

The importance of the composition of participants in the learning network, across professional and organizational levels was emphasized, both by the municipality and hospital participants. The participants also described how the learning network had brought stimuli in from the national level, and they described how they had engaged in national networks, bringing their experiences from the local learning network into the broader context. These interactions, internal, across organizational levels, and even nationally, led to a feeling of competence, a satisfaction about having fresh knowledge, and a feeling of being able to handle changes and new guidelines.

Participant 7, from municipal homecare services, explained:

*“Bringing the experiences from the learning network, we feel on top of the situation in other, national, networks”.*

Some challenges to participation were identified as being due to interference from organizational and structural frames outside the learning network. The participants from the local hospital described how the hospital administration tended to stop all travel and course-related activity for part of the year as an austerity measure. Participant 8, from the local hospital, also expressed ambivalence regarding her own motivation, leaving the normal demanding clinical day and creating a workload waiting for her return:

*“Me, as a person, I am impatient…we are trained to put out fires… I have gained a broader understanding of how to work differently…but I am not all the way there yet…”.*

### Knowledgeable understanding of a complex context

This learning network was described as adding knowledge that developed HMMs` capacity and capability for leadership based on a process understanding of their complex context. This development could be explained as reflexive processes supported by theoretical understanding and tools. The participants experienced the development of knowledge, which provided capacity for leadership. The development of common knowledge with other HMMs who they need to interact with in their normal clinical day was described as also adding capability by developing the possibility of utilizing this knowledge and developing it further to handle the complex and demanding context.

### Reflexive processes

Participation in the learning network initiated reflexive processes. These processes included reflection, a ripening process, and a flexible yet binding commitment to the network. The networks` approach to learning stimulated these reflexive processes. The learning activities were described concretely as workshops with short lessons combined with group-work. The continual repetition of central knowledge and the participants` active role in contributing to group-work and as lecturers were valued. Participant 2, from the municipal long-term care, explained:

*“The network, it is thinking work, you know, reflections…*”.

Participant 9, from the same interview, added:

*“…that it is a process…. it is something, that I have developed. You have something when you start, and then…”.*

The participants described the reflexive approach as questions asked by mentors, which initiated the participants own reflexive processes. Participant 6, from the municipal long-term care, described it like this:

*“...it gives you something to chew on further, in the clinical everyday life…”.*

A long-term commitment was described as being important to continuity, which also contributed to the development of trust among the participants. This learning network did not have an end-date. At the end of each current meeting, the participants themselves evaluated, and planned the next meeting, discussing whether and when it was needed. Participant 4, from the local hospital described the difference between committing to this network compared to a course:

*“…and that it [the learning network] is with the municipalities…. that I think is much more binding than just to be around another place…in the world because someone sent you to this place…”.*

The participants explained that the learning networks` flexible yet binding, approach made it easier to enter as new participants, but even the participants with a long-term commitment experienced the development of new knowledge. This was explained in relation to the networks approach of always building on each participant’s existing knowledge. Continuity and repetition were described as important and necessary since this type of process-work was described as demanding and time consuming.

Participant 2, from the municipal long-term care, explained:

*“…that these are things that have been repeated several times and that it is, for someone, you do not get it all, all the time, but that it is…that it is repeated…again, some of the themes…”.*

The participants described working in groups, both with participants from own organization and across organizational borders, as equally important. This importance was explained because working within and across organizational borders developed different kinds of knowledge: knowledge about internal challenges, and knowledge about interactional challenges. Sharing knowledge among the participants was in general experienced as an important approach to developing capacity and capability for leadership.

The participants from the municipalities had actively planned the periods between the network meetings and described these periods as important. The participants from the local hospital had not managed to make room for this activity but expressed that this was something they struggled to change.

The participants explained that the learning network, as a pedagogical approach, gave a meta-perspective to their clinical work place. They referred to sharing knowledge as small useful knowledge-drops collated to reflect on the shared topic. Altogether, the participants from all three focus groups compared the pedagogical approaches in the network to an education in leadership, leading to an individual ripening process.

Participant 6, from the municipal long-term care, said:

*“For me, this has been a good education in leadership, simply…”.*

In contrast, the participants described the pedagogical approaches in the learning network as unusual compared to, for instance, other leadership trainings they had attended. As participant 4, from the local hospital, explained:

*“I have thought many times that the life at the hospital should have been more like the schools we have attended… not just cut over…”.*

### Theoretical understanding and tools

The approaches in the learning network, experienced to develop HMMs capacity and capability for leadership, included a strengthening of the theoretical foundation, in close relation to practice. This foundation involved complexity theory, system theory, improvement theory, user knowledge, leadership theory, and theory about different leadership tools. The participants stated that this approach facilitated a knowledge-based practice since theory was put into relevant coherence. Several participants described their previous experiences of theoretical leadership input as fragmented.

Participant 10, from the municipal homecare services, stated:

*“…but this way of working is not…. you get in a way some tools…I feel that it has been good to get some basic knowledge and more theory, which has been useful in my job as a leader”.*

Participant 4, from the local hospital, said:

*“All the time there are knowledge drops we can bring along …Well, these are elements that make you think in a certain way, and if you take this in, it covers most of what you might need to have in your head when you are working with improvement as a leader”.*

The same participant added:

*“...but I had not had any input on my leadership [without the network], because it is all quiet in this way, there is no one who says that we have made a plan for the following years about how you could develop as a leader, no one had presented it to me, anyway…*”.

### Handling the complex and demanding context

The participation in the learning network developed the HMMs` capacity and capability by changing their every-day approach to leadership. This changed approach was based on the development of a new perspective on leadership and the development of the abilities to handle their complex and demanding contexts.

The complex and demanding context was described as a normal clinical day with no instructions. The participants explained how they were ensuring quality services, handling top-down management, and putting out fires.

Participant 1, from the municipal homecare services described it as follows:

*“Different problems where there is no blueprint, or system, which tells you how it should be”.*

The complex and demanding context was often described as being too complicated to handle. This lack of manageability lead to an identification of the self that was linked to errors and omissions. The participants described receiving this approach to leadership from their senior management, but they also shared experiences of choosing this approach themselves. With this approach, two possibilities were described if something wrong occurred: either the fault was experienced as your own, you did not manage to lead, or it had to be someone else’s fault, resulting in looking for the member of the staff who did not manage their job.

Participant 2, from the municipal long-term care, said:

*“It is easy in a way, to think, oh, I do not get it…”.*

Participant 7, from the municipal homecare services, explained:

*“It is easy to think that someone is letting us down, right…”.*

The participants explained that participation in the learning network had simplified their handling of this complex and demanding context. Or, as participant 6, from the municipal long-term care, described it:

*“It has not become easy, but it has become easier”.*

This simplified handling of the context was based on a change in the HMMs` every-day leadership, as they described it. This changed approach was experienced as a new perspective with an increased confidence in leadership. The new perspective included a different way to putting out fires and self-identifying, and it complemented their administrative and managemental skills. The participants stated that this change was achieved by the development of knowledge, process-understanding, and reflection in the learning network.

Participant 3, from the hospital said:

*“You have increased your understanding of why, if you make changes…why it does not work so fast, why things take time”.*

The changed approach included personnel management. Participant 7, from the municipal homecare services, described it as follows:

*“I think, to emphasize that the personnel must make their own choices and to try to trust their choices”.*

The changed approach also included implementing a knowledge-based practice, and consciousness about the importance of user knowledge.

Participant 2, from the municipal long-term care, stated:

*“That someone calls you and is dissatisfied with the services, and that, then you increasingly manage to take on their perspective”.*

The participants stated that the approaches from the network were implemented in practice as a more conscious priority; an approach of not looking for scape-goats, but instead searching to find the causes of the problems. They had gained a strengthened implementation capacity.

Participant 2, from the municipal long-term care, said:

*“I notice, that I have in a way lifted it from myself…. It is like now something happened that maybe should have been different, it is possible to act”.*

Participant 9, from the same interview, said:

*“…because it is not about where you let me down or where I let you down”.*

Participant 7, from the municipal homecare services, summarized this in the following way:

*“That you do not have to put out fires every time”.*

## Discussion

The purpose of this study was to identify and discuss the facilitation of HMMs` development of capacity and capability for leadership. Three focus-groups were conducted and analyzed with a critical hermeneutic foundation. In total, there were 17 participants: 16 HMMs and 1 user representative from a Norwegian learning network. We have identified two main themes: 1. Trusted interaction despite organizational and structural frames and 2. Knowledgeable understanding of a complex context.

The first theme, *Trusted interaction despite organizational and structural frames*, describes how the participants felt that the learning network gave them the opportunity to see themselves as a part of a broader perspective, the patient pathways. Participation resulted in trust in inter-professional and interdepartmental cooperation. This was contrasted with their normal fragmented and solitary day as an HMM.

The organizational and structural frames in healthcare do not emphasize inter-professional or interdepartmental cooperation, even though this is expected to occur; government, management, citizens, and central guidelines emphasize cooperation [1, 7–9]. The results of this study showed that the learning network that was studied was the only leadership related meeting point, either internally in their own organizations or across organizational borders, for the HMMs who participated. Other meetings HMMs attended were described as related to reporting, and economic management.

These organizational and structural frames exemplify what Habermas [27] explains as the system’s colonization of HMMs lifeworld. The participants had the capacity [5] for inter-professional and inter-departmental cooperation, but their capability [5] was controlled by organizational and structural frames, which prevented their interaction.

The participants were interviewed in three focus groups related to their work place. The reason for this separation was to observe if there were any differences between levels, within in a municipality, or between municipalities and hospitals. This is seen as a strength in the study design because it contributed to new knowledge that indicated that the challenges with organizational and structural frames were experienced by the hospital HMMs in particular.

In the second theme, *Knowledgeable understanding of a complex context,* the participants described their lifeworld as demanding firework, a normal clinical day with no instructions. The participants explained how they struggled to ensure qualitative healthcare while handling an overwhelming flood of concrete patient-related tasks and top-down management. This normal day is described and explored by several other studies [2, 12, 13]. This study added new knowledge by visualizing another difference between the focus groups: The participants from the municipalities had succeeded to actively plan the periods between the network meetings, while the participants from the local hospital did not manage to make room for this activity, even though this was considered important to change. These constraints, imposed by the normal clinical day in the hospital, were taken for granted, and the choices they caused were unconscious before they were communicated and reflected upon in the focus group interviews.

The results of the study provided new knowledge about handling the organizational and structural frames as a key part of HMMs` complex context. In the second theme, the participants explained how the learning network’s approaches provided knowledge and a process understanding of this complex context. These approaches were explained as the facilitation of reflection, which was supported by theoretical understanding and tools. The participants explained that these approaches contrasted the other leadership development programs they had attended, which were experienced as fragmented. These statements are supported by several previous studies, which emphasize the importance of changing the pedagogical approaches to leadership development, based on the increased complexity in healthcare [2, 3, 12, 14, 15]. This study presents new knowledge about alternative approaches, which were experienced to meet the complexity.

These alternative approaches were experienced to have initiated a holistic understanding of the demands of leadership and thereby a focus not only on increasing HMMs` capacity but also their capability to handle organizational and structural frames. The participation could thus be understood as a communicative and cooperative action undertaken by individuals and based upon mutual deliberation and argumentation. This action is facilitated by a communicative rationality, which is achieved by reflection and questioning what typically goes without question in an individually and collectively learning process [27].

The second theme provides new knowledge about how these approaches and the following learning process generated a knowledge-based practice. This development was enabled by the way in which the theoretical understanding was put into relevant coherence and facilitated by the process understanding of the complex context. This process understanding was experienced as difficult to achieve by the transactional leadership style that currently dominates healthcare [10, 40–42]. Several of the participants explained that they considered themselves as competent but that their competence was inversely related to leadership or the complex context they were a part of. The model of transformative learning [43], which added to this existing capacity and capability, chosen by this learning network was experienced as relevant and included approaches such as reflection, workshops, process work, repetition and continuity. This is, on the other hand, a learning model that is more similar to the principles of transformative leadership rather than transactional leadership [41].

The participants believed that their development of capacity and capability led to a changed approach to leadership. The changes were related to their handling of their complex reality. This is a known challenge for HMMs [1, 7, 12]. The results in the second theme add new knowledge about how the participants experience leadership with a tendency to attribute errors to specific people. This tendency was explained as having a dual nature, either participants understand the fault as their own, resulting in a feeling of failure in leadership, or they determine that it had to be someone else’s fault, resulting in looking for the member of the staff who did not manage their job. The HMMs described this strategy both as being derived from senior management and an approach they themselves made use of. Participation in the learning network had changed this approach; the HMMs explained that they had stopped looking for scape-goats. Instead, they had gained the capacity and capability to search for what caused the challenges.

The results of the study show that the participants gained confidence in leadership, and a strengthened implementation capacity, including a knowledge-based practice, and that they had extended their perspectives. The extended perspectives were particularly related to understanding services from the users` and relatives` perspectives. Process-work and reflection was developed as central elements of their leadership. The learning network could thus be described as contributing to the rationalization process, which handles the systems colonization of HMMs` lifeworld [27].

In this study, the complex context was understood in relation to both the theory of Complex Adaptive Systems [7, 19, 20] and Complex Responsive Processes [7, 23]. This theoretical perspective was found appropriate giving framework to the analysis including structures, processes, and patterns, where behavior emerges from bottom up [21]. The learning network is in this perspective an example to a meso system, in relation to the micro and macro system [44]. This study has shown that meso systems could interfere with the systems colonization of the micro systems lifeworld.

The choice of this learning network to utilize the transformative learning model [43] has influenced the results in this study, and could thus be seen as a limitation of the design. Studying other learning networks with other choices of learning models may yield different results. However, the choice of learning model was also important new knowledge added by the study, as an alternative to other learning models experienced by the participants as more typical but less functional.

Methodologically, this study’s first and second author had both participated in the network. This dual role, as both researcher and colleague of the participants, affected the study in several respects. It simplified the access to the field by building on existing trust. However, the risk of having influenced the participants` answers, is also a limitation of the design.

This study is only based on three focus groups, which gives a limited contribution to this complex context. The findings cannot immediately be generalized to other contexts. However, Kvale [34] argues that analytical generalization is a possibility, which means that the results of a study can be considered “indicative” or transferable in relation to other similar situations or settings.

This study provides new knowledge about how the choices of approaches in a learning network could facilitate HMMs` development of capacity and capability for leadership by contributing to the participants` rationalization process and thereby refuting the systems` colonization of HMMs` lifeworld. The implication for practice is a suggestion of several identified and discussed approaches to the facilitation of HMMs` development of capacity and capability for leadership, which were experienced as useful by the participants of a learning network. Further research is necessary to study how these results could be taken further out in healthcare organizations, adding knowledge to change. It would also be expedient to study the use of the networks` approaches in a clinical context, to explore if the HMMs` experiences of development are only personal or if this development influences the organization further, as experienced by personnel, users, and relatives.

## Conclusions

This learning network facilitated HMMs development of capacity and capability for leadership. The development included a combination of understanding the complex context, knowledge, trust, and confidence. The approaches in the learning network were based on transformative learning, coherence, reflection, discussion, repetition, knowledge sharing, and short lectures. These approaches can be recommended for the facilitation and support of HMMs.

## Additional file

Additional file 1: Interview guide. (DOCX 12 kb)

## Abbreviations

CAS: Complex adaptive systems
CRP: Complex responsive processes
HMM: Healthcare middle manager
NSD: Norwegian Data Protection Official for Research
