# Supplementary material for: Healthcare middle managers` experiences developing leadership capacity and capability in a public funded learning network
Source: BMC Health Serv Res. 2018 Jun 8;18:433. doi: 10.1186/s12913-018-3259-7 (PMC5994034; doi:10.1186/s12913-018-3259-7)
Supplement: Supplementary file 1 — Interview guide. (DOCX 12 kb) [file 12913_2018_3259_MOESM1_ESM.docx]

**Additional file 1**

Interview guide

How would you describe the usefulness of participating in the learning network?

How would you describe your experiences executing leadership before and after participating in the learning network?

Any changes in how you think about leadership?

Any changes in how you perform leadership?

How would you describe your abilities as a healthcare middle manager?

How is this influenced by participating in the network?

How does your participation in the network influence your staff?

How does your participation in the network influence the recipients of your services?

How can you compare these processes with other processes in your life?

Supporting questions: Can you add some examples?

Why does this happen?

How did this happen?

How did you know this?

How could this be changed.
